# Increased opioid consumption in neoadjuvant immunotherapy plus chemotherapy for patients with non‐small‐cell lung cancer: A multicenter, prospective cohort study

**DOI:** 10.1111/cns.14893

**Published:** 2024-08-04

**Authors:** Kaiyuan Wang, Jianxu Er, Yu Zhang, Chengcheng Song, Yang Yu, Ruifang Gao, Hong Xu, Xiaolin Dong, Limei Yuan, Qiangwei Liu, Jiange Han, Yonghao Yu, Yiqing Yin

**Affiliations:** ^1^ Department of Anesthesiology Tianjin Medical University Cancer Institute and Hospital, National Clinical Research Center for Cancer, State Key Laboratory of Druggability Evaluation and Systematic Translational Medicine, Tianjin's Clinical Research Center for Cancer Tianjin China; ^2^ Department of Anesthesiology Tianjin University Chest Hospital Tianjin China; ^3^ Department of Anesthesiology Tianjin Medical University General Hospital Tianjin China

**Keywords:** analgesia, neoadjuvant immunotherapy, non‐small‐cell lung cancer, opioid consumption, postoperative delirium, postoperative pain

## Abstract

**Aims:**

PD‐1 block was reported to impair opioid‐induced antinociception and affect cognitive function in rodents and non‐human primates. This prospective multicenter cohort study aims to investigate the possible impact of neoadjuvant immunotherapy with PD‐1 antibody on perioperative analgesic effect of opioids and postoperative delirium (POD) for non‐small‐cell lung cancer (NSCLC) patients.

**Methods:**

Eighty‐four NSCLC patients from three medical centers with neoadjuvant chemoimmunotherapy (nCIT) or chemotherapy (nCT) were enrolled. The primary outcome is the total perioperative opioid consumption defined as the sum of intraoperative and postoperative opioid use within 3 days after surgery. Secondary outcomes compromise of incidence of POD, pain intensity, and number of analgesic pump press. Tumor prognostic parameters and perioperative change of inflammatory cytokines and soluble PD‐L1 level were also recorded.

**Results:**

Eighty‐one patients were included in the final analysis. The total opioid consumption (sufentanil equivalent) perioperatively in the nCIT group was significantly higher than that in the nCT group, with mean difference of 60.39 μg, 95% CI (25.58–95.19), *p* < 0.001. Multiple linear regression analysis showed that nCIT was correlated with increased total opioid consumption (β = 0.305; 95% CI, 0.152–0.459; *p* < 0.001). The incidence of moderate‐to‐severe pain and cumulative analgesic pump press within 72 h was significantly higher in subjects with nCIT. There is no statistical difference in incidence of POD between groups within 72 h after surgery. The pathologic complete response rate and perioperative serum IL‐6 level were higher in the nCIT group than in the nCT group.

**Conclusion:**

Patients with NSCLC receiving nCIT warrant increased opioid consumption perioperatively and suffered from more postoperative pain.

**Clinical Trial Registration:**

NCT05273827.

## Graphical abstract

Patients with non‐small‐cell lung cancer receiving neoadjuvant chemoimmunotherapy warrant increased opioid consumption perioperatively and suffered from more postoperative pain compared with patients receiving neoadjuvant chemotherapy alone. However, there is no statistical difference in incidence of postoperative delirium (POD) between groups within 72 h after surgery.

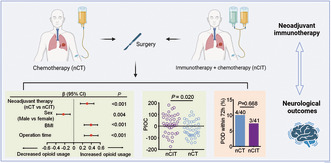

## 1 INTRODUCTION

Lung cancer is the second most commonly diagnosed cancer and the leading cause of cancer death in 2020 worldwide,^1^ with 87% of cases classified as non‐small‐cell lung cancer (NSCLC). The treatment for NSCLC underwent transformation through the advent of targeted therapies and the emergence of immune checkpoint inhibitors (ICIs), particularly monoclonal antibodies against PD‐1 and PD‐L1.^2^, ^3^ For patients with resectable NSCLC, recent studies indicate that the addition of neoadjuvant PD‐1 antibodies therapy before surgery can markedly enhance postoperative survival, when compared with chemotherapy alone,^4^, ^5^, ^6^ which showed an extensive application of PD‐1 antibody in surgical patients.

Meanwhile, prior investigations have revealed that PD‐1 is also expressed in dorsal root ganglia (DRG) sensory neurons in both mice and humans.^7^ Blocking PD‐L1/PD‐1 pathway with ICIs induced mechanical and spontaneous pain in murine melanoma models.^8^ Subsequent research has demonstrated the co‐expression of PD‐1 and Mu‐opioid receptors (MOR) in DRG neurons, implicating PD‐1 is required for the analgesic effects of opioids such as morphine. Anti‐PD‐1 therapy impaired the analgesic efficacy of opioids in mouse and non‐human primates.^9^ Thus, for patients receiving neoadjuvant anti‐PD‐1 therapy, whether the analgesic effect of opioid be affected perioperatively warrants further exploration.

In our previous single‐center retrospective study, we have reported that neoadjuvant immunotherapy is associated with increased total amount of perioperative opioid in patients with esophageal cancer undergoing esophagectomy.^10^ The current prospective multicenter study aimed to further verify the potential influence of PD‐1 treatment on perioperative opioid consumption and postoperative pain intensity. Since PD‐L1/PD‐1 pathway was reported to modulate the cognitive function and learning and memory ability of mice and non‐human primates,^7^, ^11^, ^12^ we also evaluated the incidence of postoperative delirium (POD) in the present study.

## 2 METHODS

### 2.1 Study design

This multicenter prospective cohort study was approved by the ethical committee of Tianjin Medical University Cancer Institute and Hospital, Tianjin Medical University General Hospital, and Tianjin University Chest Hospital, China. This observational study was registered in Clinical Trials (NCT05273827) on October 7, 2021. All participants provided written informed consent.

### 2.2 Subjects

We screened patients aged 18 years or older undergoing curative surgery for lung cancer with neoadjuvant chemoimmunotherapy (nCIT) or neoadjuvant chemotherapy alone (nCT). Inclusion criteria comprised the following: histologically confirmed stage II or stage IIIA NSCLC and meet the requirements for R0 resection; American Society of Anesthesiologists (ASA) status I–III; body mass index (BMI) 18.5–30; normal function of coagulation and vital organs such as heart, lungs, kidney, and liver; and eligible to receive platinum‐containing two‐drug chemotherapy. Exclusion criteria were as follows: prior cancer treatment including chemotherapy, radiation, or targeted therapy; preoperative opioid analgesia; severe chronic or active infection requiring systemic antibacterial, antifungal, or antiviral therapy, including tuberculosis infection; history of antipsychotic medication in the last 6 months; and preoperative Mini‐Mental State Examination (MMSE) score <23.

Patients in the nCT cohort underwent platinum‐based chemotherapy. The chemotherapy protocol included paclitaxel plus cisplatin for squamous cell carcinoma and pemetrexed plus cisplatin for adenocarcinoma. Patients in the nCIT cohort received intravenous administration of anti‐PD‐1 monoclonal antibody combined with the above platinum‐based chemotherapy. All patients underwent 2–4 cycles of neoadjuvant treatment, with each cycle lasting 21 days. Surgery was performed 4–6 weeks after the last neoadjuvant therapy.

### 2.3 Anesthesia and postoperative analgesia

MMSE was performed 1 day before the surgery. Anesthesia induction involved intravenous administration of midazolam (0.15 mg kg^−1^), etomidate (0.2 mg kg^−1^), rocuronium (0.9 mg kg^−1^), and sufentanil (0.4–0.6 μg kg^−1^).^13^ Anesthesia was maintained with continuous infusion of propofol to maintain the bispectral index between 40 and 60, and remifentanil (0.1–0.3 μg kg^−1^ min^−1^) titrated to maintain blood pressure and heart rate to within 20% of their values before skin incision. Rocuronium was administered intermittently as needed. Fluid infusion and blood transfusion were provided according to routine practice. Propofol and remifentanil infusions were halted during skin closure, and sufentanil (0.1–0.3 μg kg^−1^) was administered. Surgical wound infiltration was performed with ropivacaine at the end of surgery. The endotracheal tube was removed when the patient exhibited adequate spontaneous breathing and recovery of consciousness. They were transferred to the postanaesthesia care unit (PACU) for further monitoring and then back to the postoperative intensive care unit (PICU) of surgical wards.

Postoperative analgesia utilized patient‐controlled analgesia (PCA) with the following formulation: sufentanil 3 μg kg^−1^, dexmedetomidine 3.75 μg kg^−1^, and 0.9% sodium chloride solution diluted to 100 mL. The background infusion rate was set at 1 mL/h, and the boluses volume was 1 mL with a 15‐min lockout interval. The assessment of pain intensity was conducted using the score of Numerical Rating Scale (NRS). PCA application extended for 72 h postoperatively, ensuring that the patient's NRS score remained ≤3. If the NRS score was ≥4, intramuscular morphine 10 mg was administered as rescue analgesia. Postoperative nausea and vomiting were treated with intravenous ondansetron.

### 2.4 Primary outcome

The primary outcome is the total perioperative opioid consumption defined as the sum of intraoperative opioid use and postoperative opioid use including opioid administrated in the PACU and PICU or as rescue analgesia within 3 days after surgery. The amount of remifentanil and morphine were converted into sufentanil equivalent dose for statistical analysis (remifentanil 100 μg = morphine 10 mg = sufentanil 10 μg).^14^, ^15^, ^16^

### 2.5 Secondary outcomes

Secondary outcomes compromise of NRS score at rest and with movement before surgery, immediately after surgery, and at 24, 48, and 72 h after surgery; no. of total effective PCA presses at postoperative 24, 48, and 72 h and the time of first PCA press; percent of patients with moderate‐to‐severe pain within 72 h (any NRS pain score of 4 or higher); intraoperative opioid consumption; and postoperative opioid consumption.

The occurrence of postoperative delirium (POD) was also assessed using the 3D‐CAM during the first 3 days postoperatively.^17^ Patients were followed up at 16:00–18:00 in the afternoons each day to obtain the above outcomes including NRS score, PCA presses, and incidence of POD.

### 2.6 Other outcomes

Blood samples were collected from patients before surgery, at the end of surgery, and 24 h postoperatively for the analysis of serum levels of IL‐6, IL‐10, TNF‐α, and soluble PD‐L1 (sPD‐L1). The ELISA kit for IL‐6 (CSB‐E04638h), IL‐10 (CSB‐E04593h), TNF‐α (CSB‐E04740h), and sPD‐L1 (CSB‐E13644h) was purchased from CUSABIO. ELISA was conducted in accordance with the manufacturer's instructions. A standard curve was performed for each experiment.

Dosage of propofol and tumor prognosis parameters including pathologic complete response (pCR) rate (0% viable tumor in resected lung and lymph nodes) and ratio of R0 resection (surgical margin is microscopically negative for residual tumor) were also recorded.

### 2.7 Statistics and data analysis

IBM SPSS Statistics version 26 (IBM, Armonk, NY) and GraphPad Prism 6.0 (GraphPad Software, San Diego, CA) were employed for statistical analysis and figure production. Normality was evaluated by the Shapiro‐Wilk statistic. Continuous variables are presented as mean with standard deviation or median with 25th and 75th percentiles (IQR) as appropriate. Numerical data were compared using two‐sample *t*‐test or Mann‐Whitney *U*‐test with median differences and 95% confidence intervals (CIs) estimated with the Hodges‐Lehmann method. Categorical variables were assessed by Fisher's exact test or chi‐square test. Univariable liner regression analysis was used to detect factors associated with the primary outcome (total opioid consumption). The screened factors were further taken into the multivariable liner regression analysis to explore their association with primary outcome and calculate the standardized coefficients (β value).

As exploratory analyses, we calculated the area under the curve (AUC) of NRS sore and the pain intensity and opioid consumption (PIOC) within 72 h after surgery according to the reported proposals.^13^, ^18^ The AUC of NRS could outbalance uneven measurement intervals, providing an integration of pain intensity versus time. PIOC further delivered a more precise portrayal of the dynamic characteristics of postoperative pain and the efficacy of analgesic drugs and mitigated the potential for exaggerated statistical significance. The range of the PIOC is −200% to 200%. Values above 0 indicate increased summed AUC and opioid consumption. The between‐group differences were compared with the Mann–Whitney *U*‐tests, with median differences and 95% CIs calculated using the Hodges‐Lehmann estimators. All statistical tests were two‐sided, and 0.05 was set as the level of significance.

### 2.8 Sample size estimation

According to the polit study, to detect a difference of 20% of total perioperative opioid consumption, with a ratio of 1:1 in either group, set α = 0.05 and β = 0.2, there will be 38 patients in each group via PASS 15.0. Considering a loss of visit of 10 percent and to increase the statistical power, 42 patients in each group were enrolled.

## 3 RESULTS

### 3.1 Study characteristics

**FIGURE 1:**
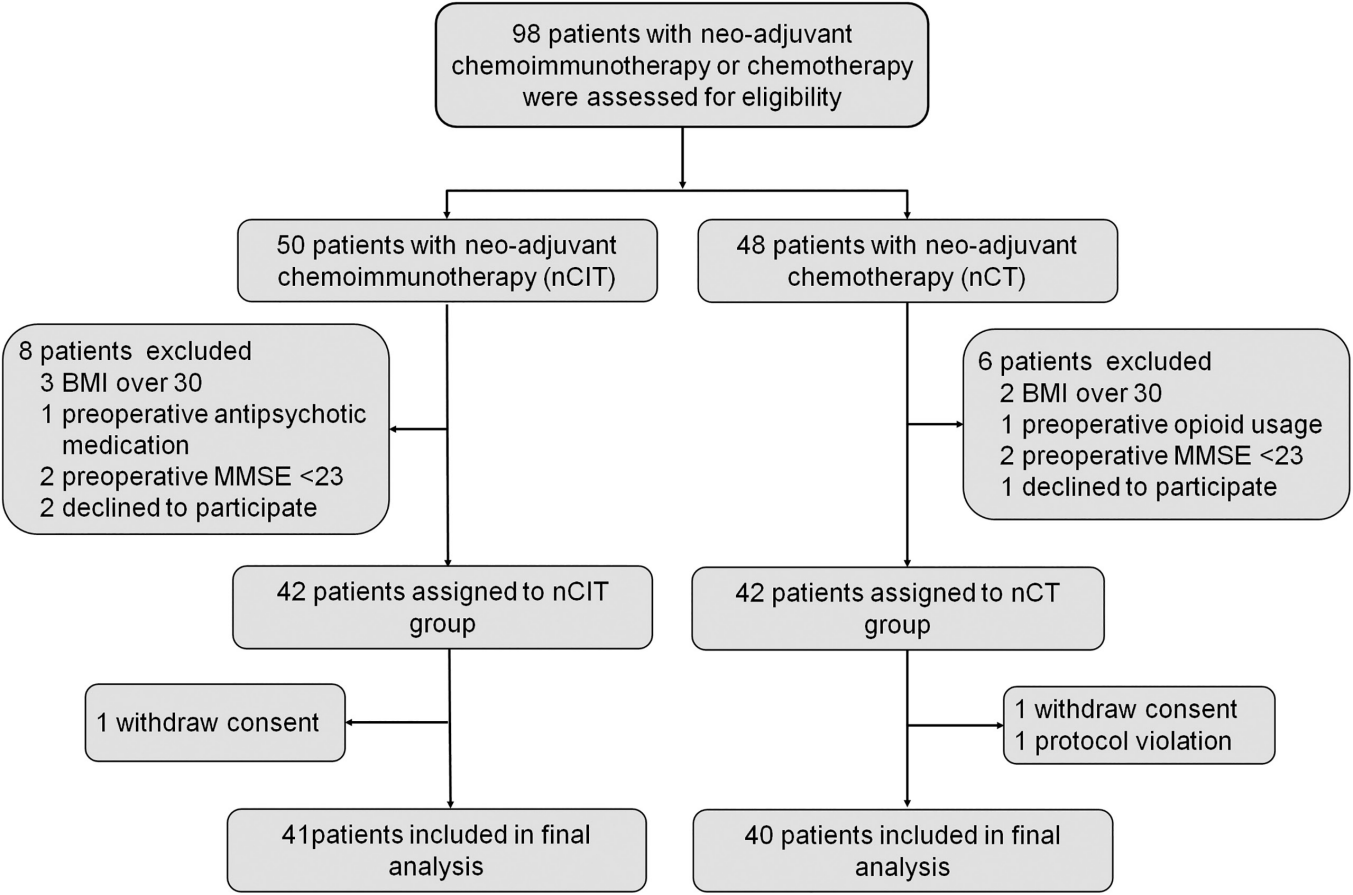
Flow chart of the study. MMSE, the Mini‐Mental State Examination.

Between March 2022 and December 2023, 98 NSCLC patients with neoadjuvant chemoimmunotherapy or chemotherapy were assessed for eligibility. Eighty‐four patients were further enrolled and assigned to the nCIT cohort (*n* = 42) and nCT cohort (*n* = 42) according to their neoadjuvant therapy. During the study, three subjects were further excluded due to withdrawing consent or proposal violation. Therefore, 81 patients were included in the final analysis (Figure 1).

The two groups were well balanced on baseline characteristics. There was no statistical difference in the general information including age, sex, BMI, history of smoking and alcohol, ASA status, Charlson comorbidity index (CCI) score, education years, preoperative MMSE, and preoperative parameters for lung, liver, kidney, coagulation, and cardiovascular function (Table 1).

**TABLE 1:** Baseline characteristics.

Items	nCIT group (*n* = 41)	nCT group (*n* = 40)	*p*‐value
Age (year)	65 (60, 70)	65 (59, 67)	0.212
Sex (*n*/%)			
Male	33 (80.5)	27 (67.5)	0.182
Female	8 (19.5)	13 (32.5)	
BMI	24.62 (3.76)	25.02 (2.98)	0.598
ASA stage			
I	4 (9.76)	4 (10)	0.386
II	21 (51.22)	26 (65)	
III	16 (39.02)	10 (25)	
Smoking (*n*/%)	32 (78.05)	27 (67.5)	0.286
Alcohol (*n*/%)	22 (53.66)	18 (45)	0.436
Charlson comorbidity index score	0 (0, 0)	0 (0, 3.75)	0.186
Ischemic stroke (*n*/%)	2 (4.88)	3 (7.5)	0.676
Hypertension (*n*/%)	18 (43.9)	16 (40)	0.722
CAD (*n*/%)	3 (7.32)	3 (7.5)	1
Diabetes (*n*/%)	5 (12.20)	5 (12.5)	1
Education years	9 (8, 9)	9 (5, 12)	0.501
Preoperative MMSE	26 (25, 27.5)	26 (25, 28.75)	0.448
Laboratory test results			
Hemoglobin (g/L)	136.6 (17.4)	129.2 (17.6)	0.062
Albumin (g/L)	42.4 (4.54)	40.94 (3.5)	0.164
ALT (U/L)	23 (18.6, 30.4)	18.5 (14, 24.75)	0.085
AST (U/L)	21 (13.9, 29.5)	24 (21.25, 30.5)	0.067
BUN (mM)	5.34 (1.60)	5.40 (1.47)	0.85
CR (μM)	72 (60, 92.6)	67.6 (56.25, 78)	0.177
K^+^ level (mM)	3.8 (3.55, 4.10)	3.8 (3.60, 4.10)	0.609
Abnormal K^+^ level (*n*/%)	7 (17.07)	3 (7.5)	0.312
Lung function			
FVC	3.3 (0.71)	3.02 (0.79)	0.094
FEV1	2.65 (0.56)	2.47 (0.64)	0.191
FEV1/FVC	80.23 (6.61)	82.51 (9.13)	0.202
MVV	90.32 (21.99)	85.44 (22.83)	0.33
DLCO SB	7.39 (1.56)	7.06 (1.45)	0.332
Coagulation (*n*/%)			
Normal	36 (87.8)	36 (90)	1
Abnormal	5 (12.2)	4 (10)	
ECG (*n*/%)			
Normal	35 (85.37)	34 (85)	1
Abnormal	6 (14.63)	6 (15)	
Echocardiography (*n*/%)			
Normal	31 (75.61)	35 (87.5)	0.253
Abnormal	10 (24.39)	5 (12.5)	

Perioperative indicators, including type of surgery, operation and one‐long ventilation time, TNM stage, histologic type, R0 resection, blood loss, fluid infusion, blood infusion, urine volume, incidence of intraoperative hypotension, arrythmia, myocardial infarction and hypoxemia, surgical complications, reoperation during hospitalization, and hospital stay, did not differ between groups. However, patients with nCIT exhibited significantly increased pCR compared to those with nCT (24.39% vs. 5%, *p* = 0.026) (Table 2).

**TABLE 2:** Perioperative parameters between two groups.

Items	nCIT group (*n* = 41)	nCT group (*n* = 40)	*p*‐value
Type of surgery			1
Open	2 (4.88)	2 (5)	
Video‐ or robot‐assisted thoracic surgery (VATS/RATS)	39 (95.12)	38 (95)	
Surgery postponed (*n*/%)	0 (0)	0 (0)	1
Operation time (min)	148 (103, 200)	130 (111.3, 158.8)	0.533
One‐lung ventilation time (min)	135 (92.5, 177.5)	120 (100, 152.3)	0.571
Propofol consumption (mg)	700 (451, 875)	755 (530, 895)	0.436
Disease stage at baseline (*n*/%)			0.409
II	32 (78.05)	28 (70)	
IIIA	9 (21.95)	12 (30)	
Histologic type (*n*/%)			0.133
Squamous	31 (75.61)	24 (60)	
Nonsquamous	10 (24.39)	16 (40)	
R0 reaction (*n*/%) (95% CI)	41 (100)	40 (100)	1
PCR (*n*/%) (95% CI)	10 (24.39)	2 (5)	0.026
Blood loss (mL)	100 (50, 100)	50 (35, 100)	0.25
Fluid infusion (mL)	1500 (1280, 1800)	1500 (1000, 1600)	0.129
Blood infusion (*n*/%)	2 (4.88)	3 (7.5)	0.676
Urine volume (mL)	300 (200, 600)	300 (200, 400)	0.17
Intraoperative hypotension (*n*/%)	12 (29.27)	9 (22.5)	0.487
Intraoperative arrhythmia (*n*/%)	2 (4.88)	3 (7.50)	0.676
Intraoperative myocardial ischemia (*n*/%)	0 (0)	2 (5)	0.241
Intraoperative hypoxemia (*n*/%)	5 (12.2)	7 (17.5)	0.502
Surgical complications (*n*/%)	3 (7.32)	2 (5)	1
Reoperation (*n*/%)	0 (0)	0 (0)	1
Hospital stay (day)	9.6 (2.2)	9.9 (3.9)	0.681

### 3.2 Primary outcome

The mean total opioid consumption perioperatively in the nCIT group was 334.4 μg, which is significantly higher than that in the nCT group (274.0 μg), with mean difference of 60.39 μg, 95% CI (25.58–95.19), *p* < 0.001 (Table 3). In the liner regression analysis, the total opioid consumption was set as the dependent variable, while sex, age, type of neoadjuvant therapy, BMI, operation time, smoking and alcohol history, and ASA stage were set as the independent variables. Results from univariable analysis showed that type of neoadjuvant therapy, sex, BMI, smoking history, and operation time were associated with the total dosage of opioid. These factors were then put into the multiple regression model, and nCIT was further proved to be associated with increased total opioid consumption (β = 0.305; 95% CI, 0.152–0.459; *p* < 0.001, Table 4).

**TABLE 3:** Primary outcome and secondary outcomes.

Outcomes	nCIT group (*n* = 41)	nCT group (*n* = 40)	Estimated effect (95% CI)	*p*‐value
Primary outcome				
Total opioid consumption	334.4 (91.59)	274.0 (62.73)	MD^†^ 60.39 (25.58 to 95.19)	<0.001
Secondary outcomes				
Postoperative delirium within 72 h (*n*/%)	3 (7.32)	4 (10)	RR 0.732 (0.175 to 3.065)	0.668
Percent of patients with moderate to severe pain within 72 h (*n*/%)	31 (75.6)	21 (52.5)	RR 0.694 (0.493 to 0.978)	0.030
Total intraoperative opioid consumption (μg)	172 (123.5, 234)	128.1 (114.1, 164)	MD 36.28 (10 to 66.75)	0.003
Average intraoperative opioid consumption (ng kg^−1^ min^−1^)	17.52 (6.26)	14.47 (4.52)	MD 3.04 (0.624 to 5.462)	0.014
Total postoperative opioid consumption (μg)	154.9 (47.57)	135.8 (30.84)	MD 19.09 (1.312 to 36.87)	0.036
Average postoperative opioid consumption (μg kg^−1^)	2.164 (0.45)	1.96 (0.37)	MD 0.20 (0.019 to 0.382)	0.031
Cumulative analgesic pump press				
24 h	1 (0, 2)	0 (0, 1)	MD 1 (0 to 1)	0.019
48 h	1 (0, 4.5)	0 (0, 2.75)	MD 1 (0 to 1)	0.108
72 h	2 (0, 2.75)	1 (0–6)	MD 2 (0 to 2)	0.032
Time to first press (h)	8.25 (2.25, 16)	18 (4.25, 22.5)	MD −9.75 (−12 to −3)	0.277
NRS score at rest				
Before surgery	0 (0, 0)	0 (0, 0)	MD 0 (0 to 0)	>0.999
0 h after surgery	1 (0, 2)	1 (0, 2)	MD 0 (0 to 0)	0.698
24 h after surgery	2 (0, 3)	2 (1, 2)	MD 0 (−1 to 1)	0.994
48 h after surgery	1 (0, 3)	1 (1, 2)	MD 0 (−1 to 1)	0.861
72 h after surgery	0 (0, 1.5)	0 (0, 1)	MD 0 (0 to 0)	0.509
NRS score with movement				
Before surgery	0 (0, 0)	0 (0, 0)	MD 0 (0 to 0)	>0.999
0 h after surgery	2 (0, 4)	3 (0, 3)	MD 0 (−1 to 0)	0.664
24 h after surgery	5 (3, 6.5)	3.5 (3, 5)	MD 1 (0 to 2)	0.106
48 h after surgery	4 (2.5, 5)	3 (2, 4.75)	MD 1 (0 to 1)	0.186
72 h after surgery	2 (0, 3)	2 (1, 3)	MD 0 (−1 to 0)	0.324
Exploratory analysis				
Most severe pain intensity within 72 h	5 (4, 7)	4 (3, 6)	MD 1 (0 to 2)	0.043
Area under the curve of pain intensity over 72 h after surgery				
At rest	96 (36, 168)	84 (60, 144)	MD 0 (−36 to 36)	0.805
With movement	276 (162, 330)	222 (168, 285)	MD 36 (−12 to 84)	0.183
Pain intensity and opioid consumption after surgery				
At rest	14.63 (−37.8, 85.37)	−18.29 (−46.34, 26.83)	MD 28.05 (−4.88 to 63.41)	0.085
With movement	17.07 (−50, 85.37)	−28.05 (−72.56, 39.63)	MD 43.90 (7.32 to 80.49)	0.020

Abbreviations: MD^†^, mean difference; MD, median difference; RR, relative risk.

**TABLE 4:** Factors related to perioperative opioid consumption.

	Univariable		Multivariable	
	β (95% CI)	*p*‐value	β (95% CI)	*p*‐value
Neoadjuvant therapy				
nCT (*n* = 40)	0.362 (0.153 to 0.571)	<0.001	0.305 (0.152 to 0.459)	<0.001
nCIT (*n* = 41)				
Sex				
Male (*n* = 60)	−0.339 (−0.550 to −0.128)	0.002	−0.231 (−0.384 to −0.078)	0.004
Female (*n* = 21)				
Age				
<65 (*n* = 37)	0.157 (−0.064 to 0.378)	0.162		
>=65 (*n* = 44)				
BMI	0.446 (0.246 to 0.647)	<0.001	0.391 (0.238 to 0.544)	<0.001
Smoking				
Never (*n* = 22)	0.303 (0.089 to 0.516)	0.006		
Yes (*n* = 59)				
Alcohol				
Yes (*n* = 40)	−0.007 (−0.231 to 0.217)	0.952		
Never (*n* = 41)				
ASA stage				
I–II (*n* = 55)	0.146 (−0.075 to 0.368)	0.193		
III (*n* = 26)				
Operation time	0.510 (0.318 to 0.703)	<0.001	0.396 (0.242 to 0.550)	<0.001

### 3.3 Secondary outcomes

We then compared the intraoperative and postoperative opioid consumption between the two groups separately. The results showed that both the total amount and the average dosage of opioids (taking operation time and BW into account) were significantly higher in the nCIT group than in the nCT group. The incidence of moderate‐to‐severe pain within 72 h was significantly higher in subjects with nCIT (75.6%) than in those with nCT (52.5%; relative risk 0.694; 95% CI: 0.493–0.978; *p* = 0.03). The cumulative analgesic pump press within 24 and 72 h after surgery was significantly increased in the nCIT group compared to nCT group. There was no statistical difference in the time to first press of analgesic pump. Meanwhile, the NRS score at rest or with movement demonstrated no statistical difference perioperatively (Table 3).

The incidence of POD showed a decreasing trend in the nCIT group compared with nCT group within 72 h after surgery, although there is no statistical difference (7.32% vs. 10%; RR, 0.732; 95% CI, 0.175–3.065; *p* = 0.668).

### 3.4 Exploratory analysis

**FIGURE 2:**
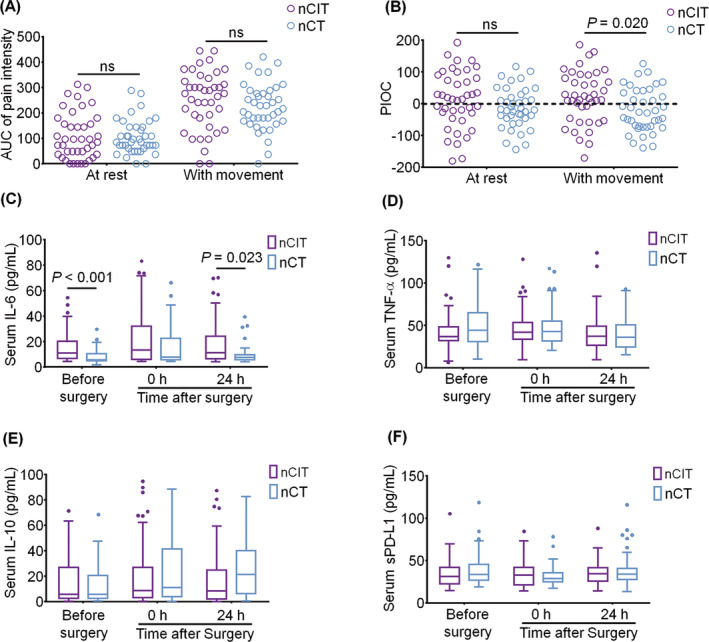
Exploratory analysis and change of cytokines perioperatively. Comparison of the area under the curve (AUC) of pain intensity sore (A) and the pain intensity and opioid consumption (PIOC) (B) between groups. Perioperative change of serum IL‐6 (C), TNF‐α (D), IL‐10 (E), and soluble PD‐L1 (F). The box and whisker plots show medians, inter‐quartile ranges, and outer ranges; individual points indicate outliers (outside 1.5 times of inter‐quartile range).

The most severe pain intensity via NRS within 72 h in the nCIT group is significantly higher than that in nCT group (median difference, 1; 95%CI, 0–2; *p* = 0.043); the difference was clinically significant (Table 3). There is no statistical difference in the AUC of pain intensity over 72 h after surgery at rest or with movement between groups (Figure 2A). The PIOC within 72 h at rest did not differ between the two groups. However, the increased PIOC within 72 h with movement was detected in the nCIT group compared to the nCT group (median difference, 43.9%; 95% CI, 7.32%–80.49%; *p* = 0.02) (Figure 2B).

### 3.5 Inflammatory cytokines and serum sPD‐L1 levels

The serum IL‐6 level was higher in the nCIT group than that in the nCT group before surgery (median difference, 3.463; 95% CI, 1.439–7.726; *p* < 0.001) and at 24 h after surgery (median difference, 2.883; 95% CI, 0.330–6.741; *p* = 0.023) (Figure 2C). There was no statistical difference in the serum level of TNF‐α, IL‐10, and sPD‐L1 perioperatively (Figure 2D–F).

## 4 DISCUSSION

ICIs, mainly monoclonal antibodies blocking PD‐1 and its ligand PD‐L1, have revolutionized the therapeutic landscape for NSCLC, particularly in the locally advanced (stage III) and metastatic (stage IV) settings.^19^ For resectable NSCLC, surgical intervention remains the primary therapeutic approach; however, 30 to 55% of patients experience disease recurrence and succumb to their illness after surgery, necessitating neoadjuvant therapies like chemotherapy or radiotherapy.^20^, ^21^ Neoadjuvant immunotherapy is an emerging concept designed to enhance host immunity against existing tumor neoantigens and eliminate micrometastatic disease prior to clinical or radiological manifestation. This strategy has demonstrated success across various solid tumor types.^22^ For patients with resectable NSCLC, several phase 3 trails have proved that neoadjuvant PD‐1 antibody plus chemotherapy resulted in prolonged event‐free survival and a higher percentage of patients with a pCR than chemotherapy alone.^4^, ^6^ The addition of PD‐1 treatment to neoadjuvant chemotherapy did not escalate adverse event incidence or compromise surgical feasibility.^22^ In the current study, we also found the increased PCR in nCIT patients compared with patients with nCT, which indicate the superior antitumor effect of this regimen.

Meanwhile, recent studies revealed the widespread expression of PD‐1 within the nervous system, including neurons and glial cells in DRG, spinal cord, and brain, where it regulates neural signals associated with pain, functioning as a neural checkpoint.^7^, ^23^, ^24^, ^25^ PD‐1 was also proved to be co‐expressed with MOR in both murine and human DRG neurons, and knocking out PD‐1 diminishes the inhibitory effects of morphine on calcium channels and reduces release of presynaptic excitatory neurotransmitter. The administration of anti‐PD‐1 antibody to mice and non‐human primates enhances opioid‐induced hyperalgesia and tolerance while markedly suppressing the analgesic effects of opioids.^9^ Thus, it is possible that anti‐PD‐1 treatment may impair the analgesic effect of opioid in cancer patients. Previously, we retrospectively enrolled 351 patients with esophageal cancer receiving nCT or nCIT for esophagectomy and found that the addition of immunotherapy could significantly increase the overall perioperative opioid demand.^10^ In this prospective multicenter cohort study, we further verified the association of nCIT and increased opioid consumption in patients with NSCLC. We also demonstrated the incidence of postoperative moderate‐to‐severe pain, and the number of PCA press is higher in patients with nCIT than in nCT group. Taken together, it is crucial to monitor pain intensity closely and provide adequate analgesia for patients with nCIT perioperatively.

Anti‐PD‐1 antibodies were reported to trigger the release of inflammatory cytokines including IL‐6, TNF‐α, and IL‐10 from various immune cells.^26^, ^27^ In the present study, we also detected the increased serum level of IL‐6 in the nCIT group compared with that in the nCT group. As a pro‐inflammatory cytokine, IL‐6 engages in neuropathic pain and cognitive impariment.^28^, ^29^, ^30^, ^31^ The increased IL‐6 level from anti‐PD1 therapy could also explain the increased pain and opioid consumption. In the present study, we did no find difference in the incidence of POD which is a major cognitive disorder for thoracic patients^32^ between the nCIT group and nCT group. PD‐L1/PD‐1 pathway was reported to regulate the cognitive function and learning and memory ability of mice and non‐human primates,^7^, ^11^ demonstrating the neuronal protective role of PD‐1 blockade. The current result may reflect the complex modality when encountering anti‐PD‐1 treatment to the postoperative cognitive function.

This study has limitations that should be acknowledged. Firstly, we did not include patients treated with PD‐L1 inhibitors. Compared with anti‐PD‐1 treatment, whether neoadjuvant PD‐L1 blockade leads to similar impact on pain and opioid requirement needs further investigation. Secondly, the potential impact on chronic post‐surgical pain needs to be observed during the long‐term follow‐up.

## 5 CONCLUSIONS

Patients with NSCLC receiving nCIT benefit from elevated pCR but warrant increased opioid consumption perioperatively and suffered from more postoperative pain. Multimodal analgesia strategies with close pain monitoring should be taken to ensure adequate and safe analgesia for these patients.

## AUTHOR CONTRIBUTIONS

KW: conceptualization; data curation; formal analysis; and writing—original draft. JE: investigation; data curation; and methodology. YZ: data curation; formal analysis; investigation; and methodology. CS: data curation and formal analysis. YY: data curation and conceptualization. RG: data curation; investigation; and resources. HX: data curation; investigation; and resources. XD: data curation and investigation. LY: data curation and investigation. QL: data curation. JH: conceptualization; investigation; and validation. YHY: supervision and validation. YQY: conceptualization; supervision; and writing—review and editing.

## FUNDING INFORMATION

This work was funded by Tianjin Anesthesia Research Development Plan Project (TJMZ2021‐M001), Tianjin “Project + Team” Key Cultivation Program (XC202034), Tianjin Key Medical Discipline (Specialty) Construction Project (TJYXZDXK‐010A), and National Natural Science Foundation of China (82171231).

## CONFLICT OF INTEREST STATEMENT

All the authors declare no competing interests and approve the publication.

## Data Availability

All the original data or de‐identified data that support the findings of this study are available from the corresponding author upon reasonable request.
